# Home Adornment

**Published:** 1875-04

**Authors:** 


### HOME ADORNMENT.

To make home attractive and worthy to be remembered after we depart from it, to secure for it its best and most lasting influence, to make all who call it theirs—and especially the young—sorry to leave it and glad to return to it, an atmosphere of genuine comfort and taste must brood over its various appointments. The influences with which we surround our children will attend them through life. If, by the objects of ornament and art with which we encircle them, we beget in their minds a taste for the beautiful, that taste never departs. We can not measure the effects of early influences, surroundings, and associations. For good or for evil, they color our whole after life. The son carries from his father's house good or bad tastes, good or bad habits, good or bad temper, and by and by his own home feels the result. A generation later, other young families are organized with their heritage of gentleness or vulgarity.

We have been reflecting upon the importance of doing all in our power for the adornment of our homes, in view of the influence which is thus exerted, having been led into this train of thought by a visit lately paid to the great furniture house of Messrs. DeGraffe & Taylor, No. 152 West Twenty-Third Street. Here may be seen every article of household use and adornment which could be desired in any home, however refined, together with a great supply of the less expensive though equally essential goods, all most thoroughly made. We can cordially recommend this worthy house to all our readers.

